# Can Large Language Models Aid Caregivers of Pediatric Cancer Patients in Information Seeking? A Cross‐Sectional Investigation

**DOI:** 10.1002/cam4.70554

**Published:** 2025-01-07

**Authors:** Emre Sezgin, Daniel I. Jackson, A. Baki Kocaballi, Mindy Bibart, Sue Zupanec, Wendy Landier, Anthony Audino, Mark Ranalli, Micah Skeens

**Affiliations:** ^1^ The Abigail Wexner Research Institute at Nationwide Children's Hospital Columbus Ohio USA; ^2^ The Ohio State University College of Medicine Columbus Ohio USA; ^3^ Centre for Health Informatics Australian Institute of Health Innovation Macquarie University Sydney Australia; ^4^ Division of Hematology/Oncology Nationwide Children's Hospital Columbus Ohio USA; ^5^ Hematology/Oncology Department Hospital for Sick Children (Sick Kids) Toronto Ontario Canada; ^6^ Institute for Cancer Outcomes and Survivorship, School of Medicine University of Alabama at Birmingham School of Medicine Birmingham Alabama USA

**Keywords:** artificial intelligence, health care communication, health literacy, large language models, patient education, pediatric oncology

## Abstract

**Purpose:**

Caregivers in pediatric oncology need accurate and understandable information about their child's condition, treatment, and side effects. This study assesses the performance of publicly accessible large language model (LLM)‐supported tools in providing valuable and reliable information to caregivers of children with cancer.

**Methods:**

In this cross‐sectional study, we evaluated the performance of the four LLM‐supported tools—ChatGPT (GPT‐4), Google Bard (Gemini Pro), Microsoft Bing Chat, and Google SGE—against a set of frequently asked questions (FAQs) derived from the Children's Oncology Group Family Handbook and expert input (In total, 26 FAQs and 104 generated responses). Five pediatric oncology experts assessed the generated LLM responses using measures including accuracy, clarity, inclusivity, completeness, clinical utility, and overall rating. Additionally, the content quality was evaluated including readability, AI disclosure, source credibility, resource matching, and content originality. We used descriptive analysis and statistical tests including Shapiro–Wilk, Levene's, Kruskal–Wallis *H*‐tests, and Dunn's post hoc tests for pairwise comparisons.

**Results:**

ChatGPT shows high overall performance when evaluated by the experts. Bard also performed well, especially in accuracy and clarity of the responses, whereas Bing Chat and Google SGE had lower overall scores. Regarding the disclosure of responses being generated by AI, it was observed less frequently in ChatGPT responses, which may have affected the clarity of responses, whereas Bard maintained a balance between AI disclosure and response clarity. Google SGE generated the most readable responses whereas ChatGPT answered with the most complexity. LLM tools varied significantly (*p* < 0.001) across all expert evaluations except inclusivity. Through our thematic analysis of expert free‐text comments, emotional tone and empathy emerged as a unique theme with mixed feedback on expectations from AI to be empathetic.

**Conclusion:**

LLM‐supported tools can enhance caregivers' knowledge of pediatric oncology. Each model has unique strengths and areas for improvement, indicating the need for careful selection based on specific clinical contexts. Further research is required to explore their application in other medical specialties and patient demographics, assessing broader applicability and long‐term impacts.

## 1 Introduction

Pediatric oncology presents unique challenges in treatment and the need for accurate caregiver information and education during the course of therapy. Caregivers often seek detailed information regarding their child's condition, treatment, and side effects [1]. The need for effective real‐time information is critical as it impacts the caregivers' understanding of cancer treatment and ability to make informed decisions to support their child through the treatment journey. The literature on pediatric cancer caregiver information‐seeking behaviors reports a significant number rely on informal sources (e.g., the internet) for health‐related questions [2]. One study reports up to 87% of oncology caregivers frequently rely on search engines (e.g., Google search) and similar tools (e.g., chatbots) because of convenience and high accessibility [3, 4]. Traditional information sources (e.g., pamphlets or booklets), although valuable, may not always provide the tailored feedback caregivers seek [5, 6]. This can create a challenging environment of limited resources and timely answers for unique caregiver concerns. Intelligent systems could bridge the gap by providing just‐in‐time and tailored responses [7], such as personalized nutrition or medication feedback [8, 9], explanation of treatment plans and access to evidence‐based guidelines [10, 11].

Artificial intelligence (AI) models (such as large language models [LLMs]) demonstrate notable capabilities in natural language processing tasks in health care domain [12], surpassing previous state‐of‐the‐art methods in areas like information retrieval [13, 14, 15], question answering [16, 17, 18], and text summarization [19, 20, 21]. Particularly via publicly available tools (e.g., ChatGPT), health information seeking and access start to shift from basic keyword‐based searching to LLM‐based augmented searches. Because of their conversational interface, most of these tools are available to lay users (e.g., asking questions to ChatGPT without prompt engineering or fine‐tuning). Therefore, these tools could be valuable in pediatric oncology, providing personalized responses to caregiver queries [22]. However, it may present a risk at multiple levels, including misinformation and misguidance [23], quality [24], accuracy, and safety [17, 24]. For example, LLMs may be prone to hallucinations [25, 26] which indicates inherent assumption of the models about the expected results based on the established patterns within its knowledge (which based on the general and unvalidated information from internet‐based resources for its training dataset). Clinical care requires an exceptional level of accuracy, especially within pediatric oncology, where nuance can have life‐threatening consequences [27, 28]. Therefore, the performance of these AI‐driven tools in delivering clinically relevant, accurate, and understandable information needs to be critically evaluated. Current literature demonstrates that LLM‐based tools can address a wide array of health questions but vary in readability and accuracy based on the domain of health care [29]. To our knowledge, few studies have investigated the AI‐generated responses from the perspective of pediatric health care providers [30, 31]. Thus, the primary aim of this study was to assess AI‐supported (LLM‐supported) tools in providing useful and reliable information to caregivers of children with cancer.

## 2 Methods

**FIGURE 1:**
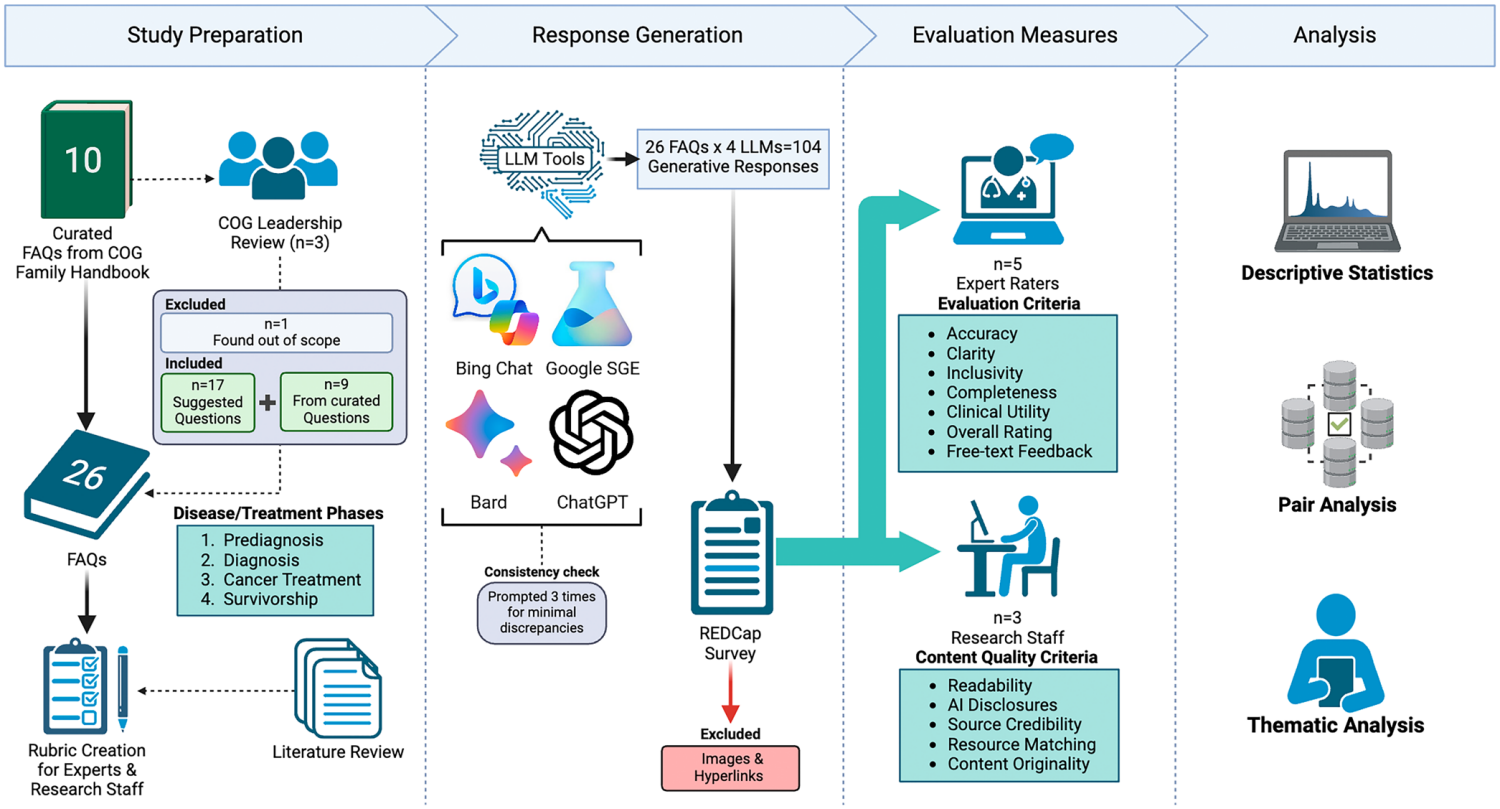
Flow diagram of study procedures.

In this cross‐sectional study, we evaluated the performance of LLM‐supported applications (see Figure 1). The responses to pediatric oncology frequently asked patient and/or caregiver questions (FAQs) [32] were assessed by a panel of five pediatric oncology experts using a comprehensive set of measures.

### 2.1 Model Selection

Publicly accessible LLM‐supported applications were selected considering their availability (free to use) and high demand/popularity. Therefore, the selection includes two LLM‐supported knowledge generation tools (Google Bard/Gemini [33], ChatGPT [34]), and two LLM‐supported search tools (Google Search with AI [SGE] [35], Bing Chat/Microsoft Copilot [36]). We will use “LLM tools” to refer to these four applications throughout this paper. Other LLM tools, such as BERT and Llama, were required deployment and tuning for optimal performance and were not technically feasible to consider within the scope of this study.

### 2.2 Question Creation

The research team members manually created a preliminary set of 10 FAQs using pediatric oncology topics from the Children's Oncology Group (COG) Family Handbook [32]. Each question was phrased to reflect the perspective of a caregiver of a child with cancer. This included rephrasing queries and follow‐up questions. A board of professional stakeholders (*n* = 3), comprised of COG leadership, reviewed the questions for plausible phrasing and topic relevance. Of the original 10 questions, one was removed as irrelevant, and nine questions were revised. The stakeholders recommended 17 additional questions to enrich the available diagnosis‐specific information grouped by disease/treatment phases. Research team combined the suggestions to create a finalized set of 26 frequently asked questions (FAQs) to prompt LLM tools (see Table 1).

**TABLE 1:** Pediatric oncology FAQs grouped by disease/treatment phase.

Phases	Questions
Pre‐diagnosis	What causes childhood cancer?
	2 Is childhood cancer contagious?
Diagnosis	3 How is childhood cancer diagnosed?
	4 What is a biopsy?
	5 How will the results of my child's biopsy be used in planning their treatment?
	6 What are the symptoms of anemia in a child with cancer?
Cancer treatment	7 What are the treatment options for my child's cancer?
	8 Can you specify different treatment options for other types of childhood cancer?
	9 What should I do if my child has symptoms of anemia?
	10 What can be done to help my child cope with hair loss?
	11 Do I need to keep my child with cancer away from other children?
	12 What causes weight gain during childhood cancer treatment?
	13 Is there anything I can do to help my child avoid excess weight gain during their treatment?
	14 What precautions should I take if my child has thrombocytopenia?
	15 My child with cancer needs a central line. What are the options?
	16 What are the pros/cons of each type of central line?
	17 What precautions do I need to take while my child is receiving chemotherapy?
	18 What should I do if my child gets a fever during cancer treatment?
	19 Will my child be able to return to school during cancer treatment?
	20 What precautions are needed if my child returns to school?
	21 What is a clinical trial?
	22 What are the pros/cons of enrolling my child on a clinical trial for treatment of their cancer?
Survivorship	23 What is the typical follow‐up plan once my child completes treatment for cancer?
	24 How long will my child continue with follow‐up after completing cancer treatment?
	25 What will be done at follow‐up visits after completing cancer treatment?
	26 Are there ways I can help my other children cope with their sibling's cancer diagnosis?

### 2.3 Expert Selection

Authors invited clinical experts over email and by word‐of‐mouth (snowball sampling). Five pediatric oncology experts volunteered as raters (two master's and doctorally prepared COG nursing leadership representatives, two oncology physicians, one doctorally prepared nurse). Total number of raters found diverse and sufficient, compared to the literature [22, 30, 31, 37]. The experts were selected on the basis of their experience in pediatric oncology and leadership within the COG. Collectively, they had an average of 20 years of experience. Among them, three had specific expertise in the care of acute leukemia, one in embryonal tumors, and one in survivorship. The group included three doctorally prepared nurses and two pediatric oncologists, with three raters holding leadership positions within COG. Each expert was introduced to the concept of generative AI and its capabilities prior to completing study activities. Then, the research team provided experts a rubric for rating tasks with instructions to train raters on the measures (see Table 2). Experts completed the rating tasks independently using a REDCap survey to organize responses [38]. On each page of the REDCap survey, an FAQ and a generative response from each LLM tool (total of 4 responses) were displayed side‐by‐side for assessment. Experts received the survey separately and were instructed to complete ratings on the basis of their clinical experience.

**TABLE 2:** Expert evaluation criteria measures and descriptions.

Measures	Question	Description	Scale
Accuracy	Is the answer an accurate reply to the question?	This criterion assesses whether the response directly and correctly addresses the question asked. An “Accurate” response fully answers the question with correct information. “Inaccurate” indicates the response is incorrect or irrelevant. “Partially accurate” suggests the response is on the right track but either contains some inaccuracies or doesn't fully address all aspects of the question. This partial rating intentionally overlaps with the measure of Completeness to better interpret how a generative response did not meet expectations.	Accurate (3), Partially Accurate (2), Inaccurate (1)
Clarity	Is the message clearly conveyed?	This measures how easily understandable the response is. “Yes” means the response is well‐structured, easy to follow, and free of jargon or ambiguity. “No” implies the explanation is confusing, poorly structured, or uses overly complex language. “Partially” indicates the response has some clarity but may be improved in certain areas for better understanding.	No (1), Partially (2), Yes (3)
Inclusivity	Is the message inclusive for a diverse range of recipients regardless of race/ethnicity, culture, etc.?	This criterion evaluates whether the response is culturally sensitive and appropriate and considers a diverse audience. A response marked as “Yes” demonstrates awareness and respect for different cultural, racial, and ethnic backgrounds, ensuring the content is relevant and appropriate for a wide range of recipients. “No” indicates that the response may be too specific to one group and might not be suitable or sensitive to the needs and perspectives of others. “Partially” suggests the response makes some effort toward inclusivity but could be improved to better address a broader audience. Example: A response that uses examples, analogies, or references which are widely recognizable across various cultures and ethnicities would score high in inclusivity. Conversely, a response heavily reliant on culture‐specific references or examples might not resonate equally with all recipients, indicating a need for improvement in this area.	No (1), Partially (2), Yes (3)
Completeness	Does the response completely answer the question?	This evaluates whether the response fully addresses all elements of the question. “Complete” indicates that the response covers all aspects of the question comprehensively. “Incomplete” means the response misses one or more critical elements of the question.	Complete (1), Incomplete (0)
Clinical utility	The response is practical in a clinical context.	This assesses how useful the response is in a practical, clinical context. It measures whether the information provided is likely to be used in consultations with patients and their families from a provider perspective. The scale ranges from “Strongly Disagree” (not useful at all) to “Strongly Agree” (extremely useful).	Strongly Agree (5), Agree (4), Neutral (3), Disagree (2), Strongly Disagree (1)
Overall rating	What is the overall score?	This rating reflects the confidence in the response's accuracy, clarity, inclusiveness, completeness, and utility. Very Low: This rating suggests effectiveness and appropriateness of the response are likely significantly different from what is perceived. It indicates major issues in accuracy, clarity, inclusivity, completeness, or utility. Low: This rating implies that there is a considerable possibility the response's actual effectiveness and appropriateness might differ significantly from what is perceived. While not entirely unreliable, the response has notable deficiencies. Moderate: This rating is used when it is believed that the response is probably close to what is perceived in terms of accuracy, clarity, inclusivity, completeness, and utility. The response is reliable but might have minor areas for improvement. High: This rating indicates a strong level of confidence in the response. It suggests that the true effectiveness and appropriateness of the response are very similar to what is perceived, showing high levels of accuracy, clarity, inclusiveness, completeness, and utility.	High (4), Moderate (3), Low (2), Very Low (1)

### 2.4 Response Generation

Four LLM tools were prompted with the complete set of FAQs in the subject domain of pediatric oncology: ChatGPT (GPT‐4 Turbo [34]), Bard (Gemini Pro patch v2023.12.06 [33]), Microsoft Bing Chat (renamed as Copilot; supported by ChatGPT‐4 and Prometheus; set to “More Balanced” mode [36]), and Google SGE (Generative AI in Search, supported by Gemini Pro, MUM, PaLM‐2 [35]) between November 30, 2023 and December 12, 2023. Each question was asked in a new session (not sequentially in a single session). Then, generated responses were copied to a text editor. Text formatting, including bold, italics, underline, highlighted, and bullet/number lists, was preserved to the greatest degree possible. Images in generative answers were excluded from expert review to standardize results to textual data. Hyperlinks were also removed to reduce bias toward the LLM tools tuned to provide links and ensure uniformity with the generated text only. To ensure LLM response consistency, the research team prompted each question three times to each LLM tool. For each LLM tool, authors (D.I.J., E.S., and B.K.) manually reviewed for discrepancies between each iteration for semantic and nuance‐level differences with medical terminology. Given minimal discrepancy (formatting, same core information for response but expressive differences), first iterations were selected for evaluation. Finally, a total of 104 generated responses by four LLM tools were selected. Responses were recorded in a REDCap form for expert evaluation. An example generated response for a question is given in Box 1. The list of generated responses can be shared upon request.

BOX 1 **Example generated response by GPT‐4 via ChatGPT (prompted on 12/2023).**

**Table:** 

**Question:** How will the results of my child's biopsy be used in planning their treatment? **Response:** The results of your child's biopsy play a crucial role in planning their treatment. Here's how the process typically unfolds: Diagnosis Confirmation: The primary purpose of a biopsy is to confirm a diagnosis. Depending on what the doctors suspect (like a tumor, infection, or other condition), the biopsy will reveal the exact nature of the issue. Determining the Type and Stage: Especially in the case of cancer, a biopsy can determine not just the presence of cancerous cells, but also the specific type and stage of the cancer. This information is vital for deciding the most effective treatment approach. Tailoring Treatment Plans: Treatment plans are often customized based on the biopsy results. For instance, certain types of cancer respond better to certain medications, and the choice of chemotherapy or radiation therapy, if needed, often depends on the biopsy findings. Identifying Genetic Markers: Some biopsies can reveal genetic markers that are significant for treatment. In cancer, for example, certain genetic mutations might make the cancer more susceptible to specific drugs. Monitoring Response to Treatment: In some cases, repeat biopsies are performed during or after treatment to see how the disease is responding. This helps in adjusting treatment plans if necessary. Guiding Surgical Decisions: If surgery is a treatment option, the biopsy results can help in planning the extent of surgery required. This is particularly true in cancer treatment, where the goal is often to remove all cancerous tissue. Remember, the exact use of biopsy results can vary depending on the specific medical condition and individual circumstances. It's important to discuss with your child's health care team how the biopsy results will inform their particular treatment plan.

### 2.5 Evaluation Criteria and Measures

Two groups of evaluation measures were created to assess the performance of the LLM tools. (1) Expert evaluation criteria include accuracy, clarity, inclusivity, completeness, clinical utility, and overall rating (see Table 2 for descriptions and scales). These measures were adapted from literature investigating clinical accuracy among LLM tools [21]. Additionally, experts provided free‐text response to share feedback. (2) Content Quality Criteria include readability, presence of AI disclosure, source credibility, resource matching, and content originality [22, 39, 40, 41]. Content Quality analysis was conducted by the research team. Table 3 outlines the measures, descriptions, and sources.

**TABLE 3:** Content Quality Criteria and descriptions.

Measure	Description	Scale	References
Readability	An online health literacy editor (SHELL) was used to compute the ease of understanding of texts. This includes the Grade reading score as well as uncommon words, public health jargon, passive voice, and the use of acronyms.	5‐18th (grade of educated reading), Missing (NA)	[39]
Presence of AI disclosure	A measure of whether the generative response contains reminders for the reader to not anthropomorphize the tool, recognize the risk of misinformation, or address a medical professional for more information.	Yes (1), No (0), Not Sure (NA)	—
Source credibility	A measure of whether linked resources in a generative answer are recent (Currency), created by experts in the field (Authorship), and published transparently (Attribution) with possible conflicts of interest (Disclosure). Each of the 4 properties contributes 1 out of 4 possible points.	Not Credible (0), Partially Credible (1–3), Credible (4), Missing (NA)	[22, 42]
Resource matching	Does the linked resource support the topic described in the generative response?	Full (3), Partial (2), None (1), Missing (NA)	[40, 42]
Content originality	A measure of total words per phrase longer than 5 words in a generative answer identified by Turnitin.com to derive from another online source.	Percentage of verbatim phrases (0%–100%)	—

### 2.6 Statistical Analysis

We reported descriptive analysis, and the interrater reliability (Cohen Kappa and Weighted Kappa were calculated for pairs of experts) [43]. We anticipated occupational roles may affect interrater agreement, therefore experts were grouped into two groups to calculate paired agreement: Nurses and Physicians. In addition, statistical analyses were conducted to explore the differences between LLM tools. Expert evaluation criteria were tested for normality using the Shapiro–Wilk method and the Kruskal–Wallis *H*‐Test to report significant differences. Then we calculated the variance of homogeneity using the Levene test. Pairs of LLM tools were calculated with the Dunn test for measuring similarity between models in a matrix. IBM Statistical Package for the Social Sciences (SPSS) Statistics on Version 28.0.0.0 (190) and R version 4.3.0 (2023‐04‐21 Universal C Runtime) through RStudio 2023.12.1 Ocean Storm Build 402 were used for analysis and visuals [44, 45, 46].

## 3 Results

**FIGURE 2:**
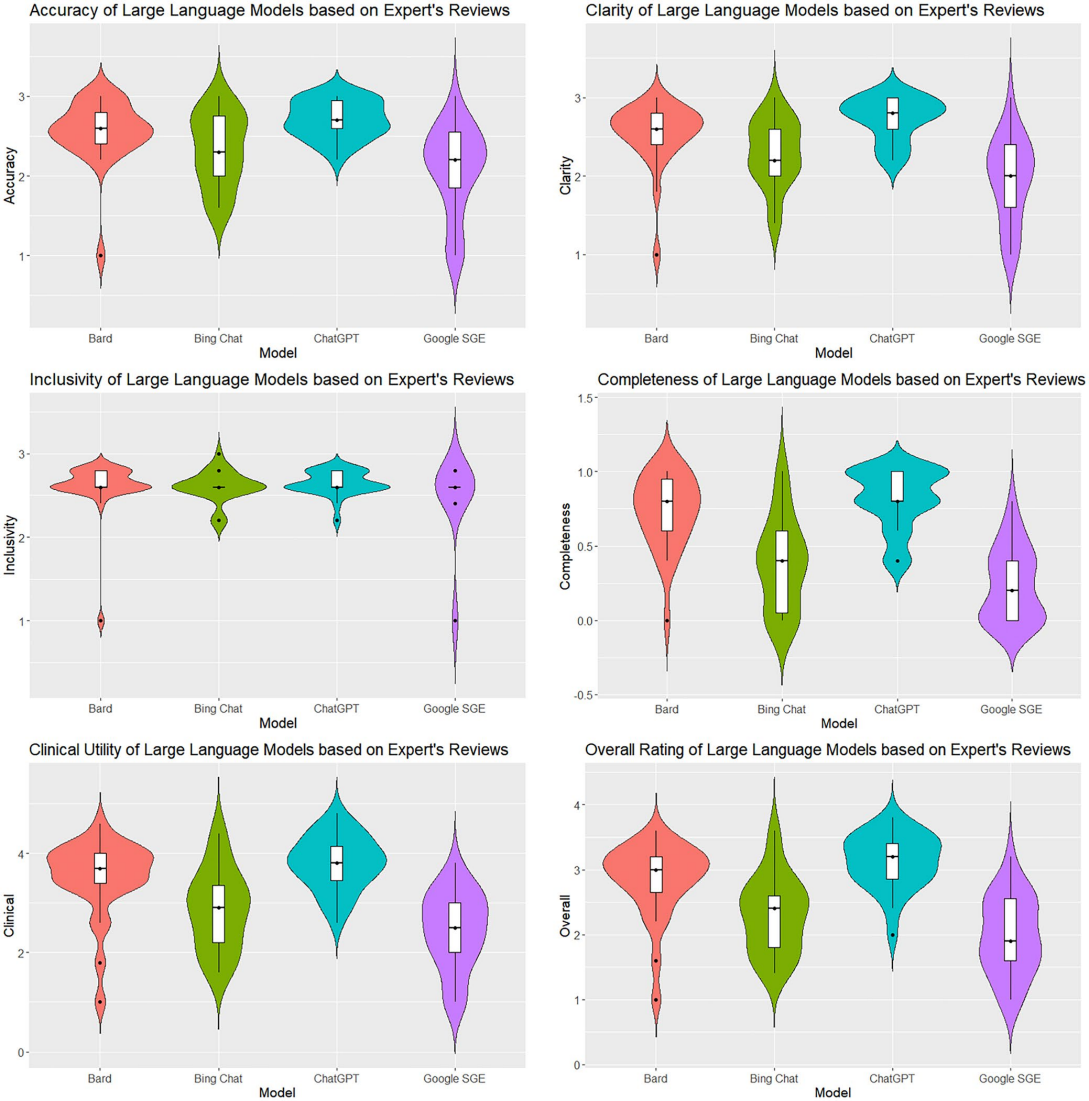
Violin graphs of expert evaluation criteria.

The expert evaluation reported the performance of four LLM tools across six measures that were rated by five pediatric oncology experts: accuracy, clarity, inclusivity, completeness, clinical utility, and overall rating (see Table 4, Figure 2). Raters demonstrated moderate agreement during the assessment (Average Cohen's *k* = 0.65). In total, the LLM tools generated 104 responses, and each expert submitted 624 ratings (with total of 3120 ratings recorded).

**TABLE 4:** LLM tools organized by expert evaluation criteria from five experts.

Model	Accuracy	Clarity	Inclusivity	Completeness	Clinical	Overall
Bard						
Mean (SD)	2.56 (0.400)	2.54 (0.411)	2.60 (0.344)	0.731 (0.246)	3.51 (0.780)	2.84 (0.582)
Median	2.60	2.60	2.60	0.80	3.70	3.00
IQR	0.40	0.40	0.20	0.40	0.60	0.60
Shapiro–Wilk Normality	0.756**	0.758**	0.437**	0.866*	0.831**	0.853*
Mean Rank	61.1	62.08	56.56	68.46	65.88	64.96
Google SGE						
Mean (SD)	2.08 (0.552)	1.95 (0.541)	2.45 (0.541)	0.239 (0.247)	2.45 (0.782)	1.98 (0.600)
Median	2.20	2.00	2.60	0.20	2.50	1.90
IQR	0.85	0.85	0.00	0.40	1.10	1.05
Shapiro–Wilk Normality	0.888*	0.943	0.505**	0.840**	0.952	0.957
Mean Rank	32.08	28.19	46.33	26.77	28.37	27.88
Bing Chat						
Mean (SD)	2.33 (0.456)	2.29 (0.424)	2.61 (0.183)	0.385 (0.309)	2.87 (0.808)	2.30 (0.586)
Median	2.30	2.20	2.60	0.40	2.90	2.40
IQR	0.80	0.60	0.05	0.60	1.20	0.85
Shapiro–Wilk Normality	0.916*	0.929	0.759**	0.898*	0.963	0.961
Mean Rank	45.46	44.06	50.40	38.56	40.81	39.33
ChatGPT						
Mean (SD)	2.71 (0.235)	2.73 (0.271)	2.65 (0.142)	0.815 (0.203)	3.81 (0.544)	3.13 (0.419)
Median	2.70	2.80	2.60	0.80	3.80	3.20
IQR	0.40	0.45	0.20	0.25	0.85	0.60
Shapiro–Wilk Normality	0.897*	0.824**	0.745**	0.803**	0.970	0.941
Mean Rank	71.40	75.67	56.71	76.21	74.94	77.83
Pair Analysis	Accuracy	Clarity	Inclusivity	Completeness	Clinical	Overall
Levene *F* Homogeneity (df) Test (Mean), *p*	4.626 (3, 100), 0.005	3.293 (3, 100), 0.026	4.272 (3, 100), 0.007	1.324 (3, 100), 0.271	1.384 (3, 100), 0.252	1.317 (3, 100), 0.273
Kruskal–Wallis H‐Test	26.451**	37.692**	2.932	49.530**	40.297**	45.382**
Dunn Test						
Bard‐Google	3.519**	4.094**	1.414	5.072**	4.498**	4.449**
Bard‐Bing	1.894*	2.177*	0.851	3.638**	3.006*	3.076*
Bard‐GPT	−1.256	−1.643	−0.021	−0.943	−1.086	−1.544
Google‐Bing	1.625	1.917*	0.564	1.434	1.492	1.373
Google‐GPT	−4.775**	−5.737**	−1.436	−6.015**	−5.584**	−5.992**
Bing‐GPT	−3.150**	−3.820**	−0.872	−4.581**	−4.092**	−4.619**

* *p* < 0.05.

** *p* < 0.001.

In terms of Accuracy, ChatGPT had the highest mean score (*M* = 2.71, SD = 0.235), followed by Bard (*M* = 2.56, SD = 0.400), Bing Chat (*M* = 2.33, SD = 0.456), and Google SGE (*M* = 2.08, SD = 0.552). The Kruskal–Wallis *H*‐test confirmed significant differences in accuracy among the models (*H* = 26.451, *p* < 0.001). Dunn's test indicated that ChatGPT was significantly more accurate than both Google SGE (*p* < 0.001) and Bing Chat (*p* < 0.01). Clarity scores were the highest for ChatGPT (*M* = 2.73, SD = 0.271), with following Bard (*M* = 2.54, SD = 0.411), Bing Chat (*M* = 2.29, SD = 0.424), and Google SGE (*M* = 1.95, SD = 0.541). The differences in clarity were statistically significant (H = 37.692, *p* < 0.001). Dunn's test showed significant clarity differences between ChatGPT and both Google SGE (*p* < 0.001) and Bing Chat (*p* < 0.01). Inclusivity scores were relatively similar across the models, with ChatGPT scoring highest (*M* = 2.65, SD = 0.142). The Kruskal–Wallis H‐test indicated no significant differences in inclusivity (*H* = 2.932, *p* = 0.403). Completeness scores were highest for ChatGPT (*M* = 0.815, SD = 0.203), followed by Bard (*M* = 0.731, SD = 0.246), Bing Chat (*M* = 0.385, SD = 0.309), and Google SGE (*M* = 0.239, SD = 0.247). Significant differences were found in completeness (*H* = 49.530, *p* < 0.001). Dunn's test revealed significant differences between ChatGPT and both Google SGE (*p* < 0.001) and Bing Chat (*p* < 0.001). Clinical Utility scores were highest for ChatGPT (*M* = 3.81, SD = 0.544). The Kruskal–Wallis *H*‐test showed significant differences in Clinical Utility (*H* = 40.297, *p* < 0.001). Dunn's test found ChatGPT significantly more clinically relevant than both Google SGE (*p* < 0.001) and Bing Chat (*p* < 0.01). Overall Rating scores were highest for ChatGPT (*M* = 3.13, SD = 0.419), followed by Bard (*M* = 2.84, SD = 0.582), Bing Chat (*M* = 2.30, SD = 0.586), and Google SGE (*M* = 1.98, SD = 0.600). The differences in Overall Rating were significant (*H* = 45.382, *p* < 0.001). Dunn's test showed ChatGPT had a significantly higher Overall Rating than both Google SGE (*p* < 0.001) and Bing Chat (*p* < 0.001).

**FIGURE 3:**
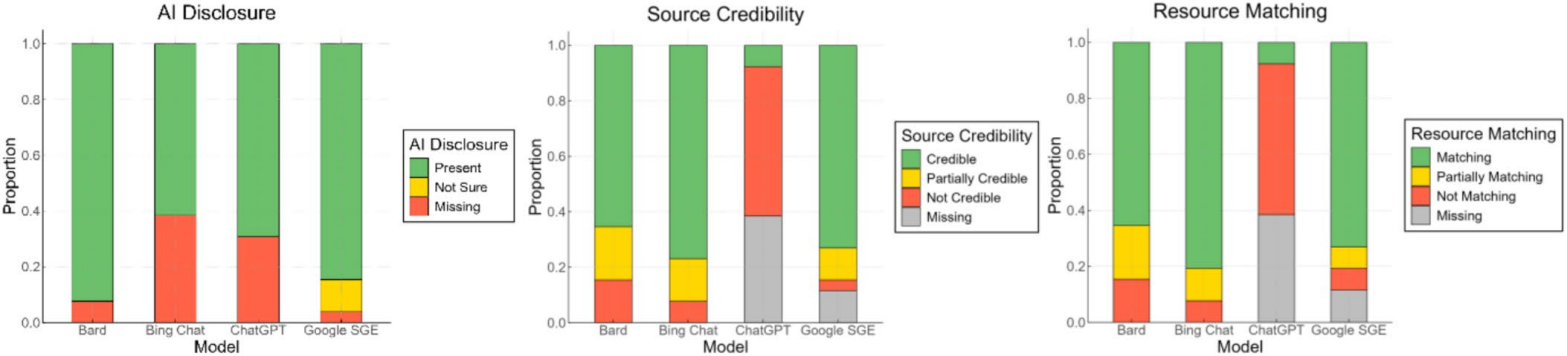
AI disclosure, source credibility, and resource matching stacked bar graph.

Content Quality results presented notable differences in LLM tool performance on AI disclosure, source credibility, and resource matching (see Figure 3). AI disclosure has been chiefly adhered to by Bard (*M* = 0.92, SD = 0.27), less by Bing Chat (*M* = 0.62, SD = 0.49) and ChatGPT (*M* = 0.69, SD = 0.46). Source Credibility was high with Bing Chat (*M* = 3.5, SD = 1.1) and Google SGE (*M* = 3.7, SD = 0.86), whereas minimally by ChatGPT (*M* = 0.5, SD = 1.32). Similarly, resource matching was highly scored for Bing Chat (*M* = 2.7, SD = 0.59) and Google SGE (*M* = 2.7, SD = 0.61), and minimally by ChatGPT (*M* = 1.3, SD = 0.66). ChatGPT (*n* = 10, 38.5%) and Google SGE (*n* = 3, 11.5%) frequently generated broken links in responses, likely due to failed embedding attempts or citing web addresses that no longer exist. ChatGPT (*M* = 0.15, SD = 0.19) and Bard (*M* = 0.14, SD = 0.19) have relatively high likelihood of producing original text compared to online resources. In contrast, Bing Chat (*M* = 0.58, SD = 0.18) showed greater tendency to produce content that closely paraphrases online resources, similar to Google SGE (*M* = 0.44, SD = 0.23).

**FIGURE 4:**
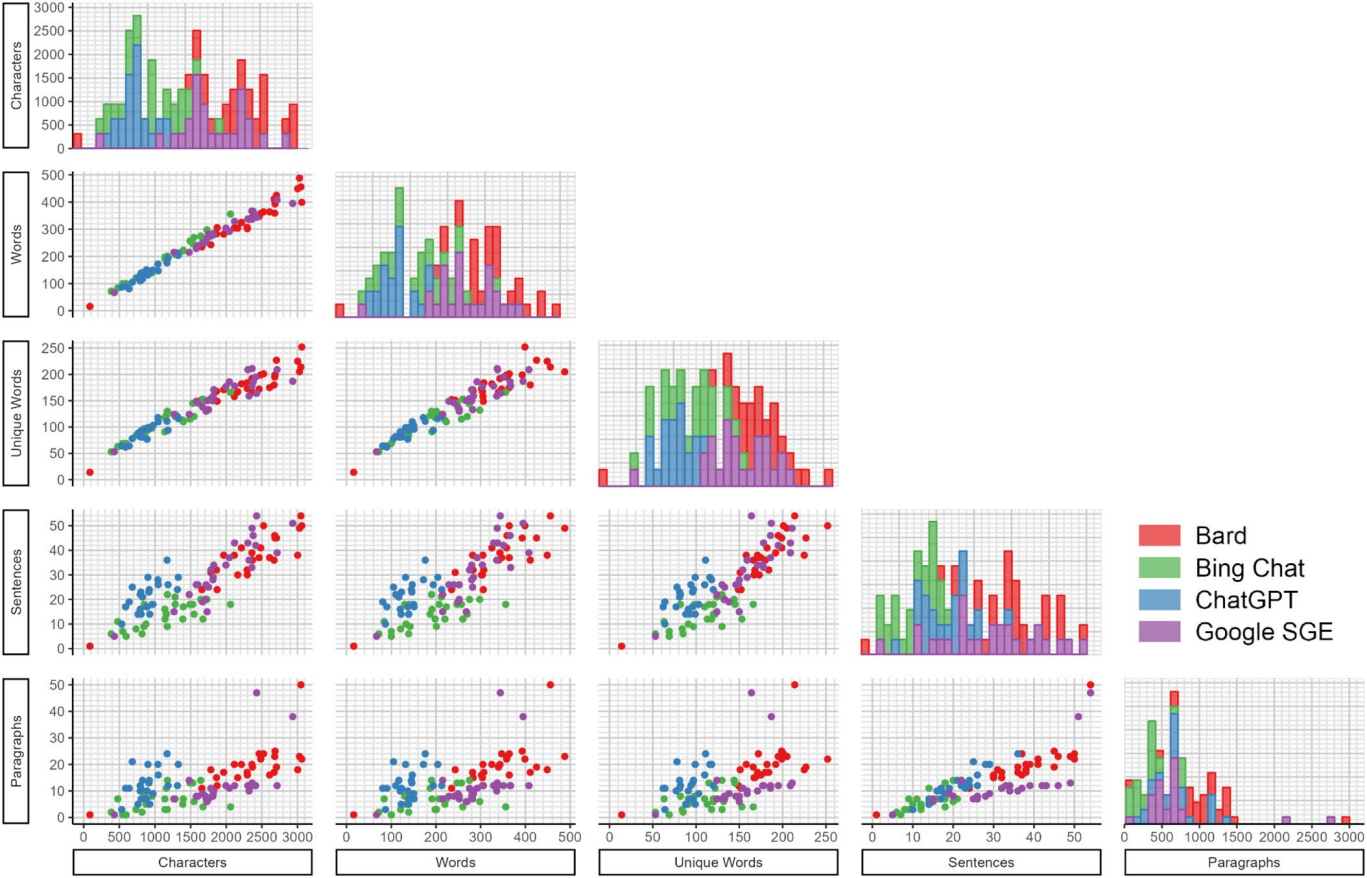
Scatterplot matrix of readability subcategories with density distributions across the diagonal bar graphs.

The models show variability in their Readability scores (see Figure 4). However, all LLM tools scored higher than 8th grade readability (which is expected for health information communications). ChatGPT often scored highest (*M* = 13.1, SD = 0.992), providing the most complex responses followed by Bard (*M* = 12.2, SD = 0.882), Bing Chat (*M* = 11.6, SD = 2.18), and Google SGE (*M* = 11.1, SD = 1.39). The Bard model tended to generate more extensive responses with more characters and words and a tendency to provide more detailed answers. On the other hand, Google SGE occasionally produced shorter responses, using lower character and word counts, possibly reflecting a more concise answering style. Bing Chat's performance was mixed, with its readability scores sometimes on the lower end, hinting at simpler or less verbose responses. Across all models, sentence structure and paragraph amount differed between LLM tools. Additionally, there is a variation in the use of unique words, highlighting differences in style and substance in the answers provided (Bard *M* = 177.1, SD = 43.0; Bing Chat *M* = 108.6, SD = 29.1; ChatGPT *M* = 159.7, SD = 34.2; Google SGE *M* = 92.2, SD = 16.9).

Finally, we reviewed 271 free‐text responses from experts and identified five themes expanding the quantitative findings: (1) accessibility of language, (2) accuracy, (3) completeness, (4) practical guidance, and (5) emotional tone and empathy. Out of five themes, Emotional Tone and Empathy was distinct from the rating rubric (please see Supporting Information).

## 4 Discussion

This study comprehensively evaluates four LLM tools—ChatGPT, Bard, Bing Chat, and Google SGE—in generating responses to frequently asked questions in pediatric oncology. Our expert analysis highlights significant differences in performance among the tools.

ChatGPT achieved the highest mean accuracy score, followed by Bard, Bing Chat, and Google SGE, respectively. Statistical analysis revealed significant differences in accuracy, with ChatGPT substantially outperforming Google SGE and Bing Chat. This aligns with prior evidence on search behaviors where ChatGPT outperforms generative search engines, including Bard [47], when answering FAQs [48]. ChatGPT provides more precise and correct information, which is crucial for caregivers seeking reliable data and for contributing to online health education [49]. Similar to accuracy, ChatGPT scored the highest in clarity and completeness, followed by Bard, whereas Bing Chat and Google SGE showed lower performance. This could result from how these LLM tools are tuned (as ChatGPT and Bard are more generalized models tuned to respond more without web search, whereas Google SGE and Bing Chat are more search oriented). However, compromising clarity to benefit quicker web search summaries may not contribute to the need for understandable information for caregivers. Yet, the nature of rapid summaries as a design philosophy may be a product of current user interactions, including caregivers, with popular search tools [50, 51, 52]. The faster, less reliable responses inherently pose a greater risk of error because of lack of clinical understanding, but they may also satisfy the target audiences' expectations. In our qualitative analysis of rater comments, emotional tone and empathy emerged distinctly from the evaluation rubric. Raters received the empathetic undertones with overall mixed view (the added warmth and support vs. being too personal and unnecessary). This resonates with the expectations from AI in health care communications, where LLM tools were interpreted as dynamic conversational partners against being augmented information sources [53, 54, 55].

Although the focus of the FAQs in this study is aligned with how pediatric oncology caregivers ask about the health care journey, personal preferences matter in the responses received. A caregiver who is looking for quick feedback or in‐depth information would likely benefit the greatest by alternating between LLM tools rather than relying on one. This behavior is already exhibited with online information seeking, where caregivers search across resources online for health care advice, including personal connections and support groups [49, 56]. This approach can be improved with LLMs, as they may provide a structure where concise responses can be expanded to provide further details and links to the relevant resources.

An important finding was LLM tools were scored above average with clinically relevant responses. More generalist models (ChatGPT and Bard) had the highest clinical utility score, compared with Bing Chat and Google SGE. This was reflected in the raters' perceived overall rating scores as well. This finding aligns with prior literature on LLM performance in the context of clinical knowledge [57]. However, significant differences in clinical utility underscore how generalized models outperform search‐oriented models. We suspect that LLM tools tuned for generalist knowledge can generate better responses compared to those tuned to web search and summarization for niche and specialized topics like pediatric oncology [58]. However, the margin of error is much narrower for health care, let alone pediatrics. Therefore, LLM tools with improved safeguards against misinformation on the internet are likely the models more suited to serve caregivers [23]. Pipelines that enable LLM tools to have access to up‐to‐date and relevant information from vetted and credible sources could mitigate risks of misinformation and hallucinations. Han et al. [59] suggests using multiple LLMs (e.g., multi‐agents) to control generative answers. Using information retrieval techniques like Retrieval Augmented Generation (RAG) with public medical indexing services, such as PubMed, could reduce the likelihood of misinformation.

We found all models were sensitive to cultural diversity and applicable to a diverse audience of caregivers. This is a promising finding toward generating inclusive content and fair representation [60]. However, given the limited set of questions used in our study, we caution that this finding may not be conclusive. More in‐depth research is needed to understand the extent of biases introduced in the LLM tools via alternative testing approaches, such as adversarial testing [61, 62, 63].

As discussed in the literature, LLM tools can help make complex information easier to understand and provide real‐time personalized education [64]. Our study found that the readability of responses from different LLM tools varied. ChatGPT often produced well‐structured, complex responses with high readability scores, suitable for more educated caregivers, but challenging for those with lower health literacy [65]. Bard generated even longer, more detailed responses, increasing unique word counts and depth but at the expense of readability. Augmented search tools like Bing Chat and Google SGE provided mixed results, sometimes offering simpler, more straightforward responses that were easier to understand but might lack detail. These overarching trends play a significant role in how caregivers should approach LLM tools in the future, as different platforms in our study demonstrated unique strengths and weaknesses. Unfortunately, all LLM tools scored far from the target 8th grade readability standard for online health information, which is most likely a larger limitation of the technology behind LLM tools [66].

### 4.1 Transparency and Reliability of LLM Tools

AI disclosure is essential to mitigate the risk of misinformation and increase transparency. This has been adopted by recent clinical tools as well, aiming to notify users that the information received is generated with AI assistance, and professional consultation might be necessary for the vital information received from these LLM tools [67]. We found that Bard consistently used AI disclosures, whereas ChatGPT rarely included in‐text AI disclosures. Augmented search tools moderately adhered to AI disclosures, adversely impacting transparency and clarity for users' understanding of the origin of information. Other LLM tools in this study (including ChatGPT) have implemented generic warnings and disclosures as either pop‐up notifications or fine prints. However, these additions should be more visible and clearly communicated.

The reliability of the responses was affected by their credibility, matching with resources shared, and content originality. Bard and Bing Chat frequently linked to recent, expert‐created resources to enhance trustworthiness. Google SGE performed moderately well, offering credible sources but with lesser consistency than Bard and Bing Chat. Similarly, aligning response content with supporting materials is essential for reliability. Bard and Bing Chat consistently linked to relevant and supportive resources, ensuring comprehensive information. Google SGE exhibited moderate performance, with occasional gaps in alignment. However, ChatGPT had lower credibility scores because of its design, which is tuned to not share resources unless explicitly prompted to, inherently affecting its performance with our metrics. In line with that, ChatGPT showed weaker performance in resource matching, again influenced by its design to avoid sharing resources, leading to a lack of alignment between content and references. Yet, ChatGPT and Bard demonstrated high content originality with low dependency on the exact syntax of the linked resources. Bing Chat, however, had a higher tendency to produce content that closely matched existing materials, followed by Google SGE. Based on the performance of these LLM tools, content originality did not necessarily equate to be reliable information (as the content source may vary). As caregivers look for information from credible resources rather than original thoughts, it might be more beneficial to use content from credible resources as‐is to convey the message as intended by the professionals. However, this also requires LLMs to ensure reliable sources while curating responses. In the future, multi‐agent approaches might be necessary to assign new LLM roles as the cross‐validator of content curated with reliable resources [68, 69, 70].

### 4.2 Implications for Personalized Patient/Caregiver Education and Clinical Practices

The application of LLMs in real‐time personalized patient education suggests a transformative shift in how caregivers' access and utilize medical information [71]. Our findings indicate that LLMs may enhance the access to personalized medical information by non‐experts. This is particularly crucial in pediatric oncology, where understanding the nuances of care and treatment options impacts decision‐making processes for caregivers [72, 73]. Real‐time access to personalized information could bridge gaps in understanding and empower caregivers, leading to more informed discussions with health care providers [74]. The ability of LLMs to provide comprehensive and accessible explanations can support the process of informed decision‐making by patients and caregivers. This is especially important in oncology, where treatment decisions can be complex and involve weighing multiple factors [75]. Furthermore, extended implementations toward personalized education ensures caregivers are aware of potential options and the associated risks and benefits. The use of LLM applications in pediatric oncology for patient/family information sharing or as educational tool is not considered to be ready for implementation. Rather, there is a need for developing guidelines, protocols, and frameworks to inform development and refinement of such tools to achieve consistent and accurate responses to caregivers and patients of pediatric oncology [23, 52].

From a clinician perspective, by automating the provision of detailed and understandable medical information, LLM‐based applications can complement clinical practices [76], and reduce the time spent responding to common queries for patients and caregivers as well as sharing evidence‐based information [77]. However, LLMs have data cutoff points, which indicates the availability of the latest information that LLMs have access to. It means that when new evidence is released after the cutoff point, it may not be available to include in LLM responses. To reduce the risk of limited information sharing, clinical applications may require the use of knowledge base to maintain up‐to‐date evidence‐based information data source [78]. The adaptability of LLMs to provide content based on specific patient contexts, as noted in the varying performance of LLMs tools, underscores the potential for personalized patient education [52].

### 4.3 Study Limitations and Future Directions

The evaluation was based on a predefined set of questions in English language, derived from the COG Family Handbook and expert input, which may only cover part of the full spectrum of caregiver inquiries. Additionally, the assessment process involved subjective evaluations by pediatric oncology experts (without patient or caregiver advocates), which could introduce bias. Moreover, the study was limited to four LLM tools only, and rapidly evolving nature of AI technology implies that the performance of LLMs may change with future updates, potentially affecting the generalizability and reproducibility of our findings over time. The concordance of experts in our study showed moderate agreement over LLM‐tool performance, but the difference may hint at opposing interpretations of the evaluation criteria. Expert ratings may have varied because of ambiguities in generative text, differences in professional background, or the rubric guidance being limited to train raters. However, interrater reliability was comparable to the literature [37, 79, 80], and readers are suggested to interpret the results considering the discrepancy (regarding mean score and standard deviations). Evaluation criteria was not validated, instead it was extended from previous assessments of LLM‐tools in the literature to meet the needs of the study methodology.

The study does not account for the dynamic interaction between patient advocates or caregivers and the LLMs. In real‐world scenarios, caregivers, advocates, or patients might ask follow‐up questions or seek clarifications and use prompt engineering, which was not simulated in this study. The static nature of our evaluation might only partially capture the conversational capabilities of the LLMs. We anticipate further research to include dynamic engagement and investigation of question‐and‐answer patterns and to explore caregiver and patient learning outcomes from diverse populations (e.g., demographics, language, and culture). Our study focused on publicly accessible LLM tools, which may not have the same capabilities as specialized or proprietary models used in clinical settings. We excluded images presented with certain LLM responses from study to mitigate unintended consequences (bias or misperception) and ensure standardization of responses across LLM responses. However, we acknowledge images can enhance content comprehension. Therefore, further research is needed to understand the multimodal feedback from LLM tools in response to caregiver questions.

The generalizability of our findings to other types of LLMs might be limited. We did not explore the integration of RAG, other knowledge base methods, or prompt engineering techniques [81], which may influence response quality. We aimed to evaluate these tools as laypersons typically use them, similar to a search engine, ensuring that the findings are relevant to real‐world scenarios where users may need more specialized knowledge in prompt engineering or access to advanced retrieval methods.

Longitudinal studies are needed to understand how using LLMs impacts caregivers over time and how caregivers' needs and LLM performance might evolve. Integrating real‐time feedback mechanisms into LLMs could enhance their adaptive learning capabilities with personalization, thereby improving the accuracy and contextual relevance of the information they provide.

## 5 Conclusion

This study contributes to the growing body of literature on the applications of AI in health care by providing a detailed evaluation of how publicly available and prominent LLM‐supported tools perform in a critical and sensitive domain. By identifying the strengths and limitations of these tools, this research offers insights about their potential role in enhancing caregivers' information access and education in pediatric oncology. Health care providers should remain cautious recommending and using LLM tools. As the technology continues to evolve, new approaches will emerge to overcome technical problems with readability and providing supporting evidence. Clinicians should advise pediatric oncology caregivers to use LLMs as supplementary information tools, emphasizing the importance of verifying information with health care providers. LLMs can offer quick, general insights and support but lack the specificity and expertise required for complex medical guidance. Ultimately, LLMs may serve as helpful adjuncts, whereas the personalized support and expertise provided by clinicians remain irreplaceable. The findings can inform future developments in AI technology, aiming to improve the accessibility and quality of health care information for caregivers and patient advocates, within pediatric oncology care and beyond in health care practices.

## Author Contributions

**Emre Sezgin:** conceptualization (lead), data curation (equal), formal analysis (equal), investigation (lead), methodology (lead), supervision (equal), validation (equal), visualization (supporting), writing – original draft (lead), writing – review and editing (equal). **Daniel I. Jackson:** data curation (equal), formal analysis (equal), methodology (supporting), project administration (lead), resources (equal), validation (equal), visualization (lead), writing – original draft (equal), writing – review and editing (equal). **A. Baki Kocaballi:** conceptualization (equal), data curation (supporting), formal analysis (equal), methodology (equal), writing – original draft (equal), writing – review and editing (equal). **Mindy Bibart:** data curation (equal), investigation (equal), writing – review and editing (equal). **Sue Zupanec:** data curation (equal), investigation (supporting), writing – review and editing (equal). **Wendy Landier:** data curation (equal), investigation (supporting), writing – review and editing (equal). **Anthony Audino:** data curation (equal), investigation (supporting), writing – review and editing (equal). **Mark Ranalli:** data curation (equal), investigation (supporting), writing – review and editing (equal). **Micah Skeens:** conceptualization (equal), investigation (equal), resources (equal), supervision (lead), validation (equal), writing – review and editing (equal).

## Ethics Statement

The authors have nothing to report.

## Conflicts of Interest

The authors declare no conflicts of interest.

## Supporting information


Data S1.

## Data Availability

The data supporting the findings of this study are available upon request from the corresponding author.
