# Supplementary material for: Can Large Language Models Aid Caregivers of Pediatric Cancer Patients in Information Seeking? A Cross‐Sectional Investigation
Source: Cancer Med. 2025 Jan 7;14(1):e70554. doi: 10.1002/cam4.70554 (PMC11705392; doi:10.1002/cam4.70554)
Supplement: Supplementary file 1 — Data S1. [file CAM4-14-e70554-s001.docx]

### Supplementary materials. Qualitative Analysis of Expert Evaluation Criteria Notes

**Methods**: The initial thematic analysis was conducted by a single researcher who employed an iterative coding process to refine themes. This process involved repeated cycles of reviewing, adjusting, and consolidating codes to ensure the themes accurately represented the scope and nuances of expert feedback. To enhance the reliability of the findings, a second researcher conducted a review of the identified themes and associated codes. Any discrepancies were resolved by consensus.

**Results**: In total, 271 free-text responses were reviewed and analyzed to identify differentiating and common themes (Table S1). We identified five themes across expert feedback regarding LLM tools, which are (1) accessibility of language, (2) accuracy, (3) completeness, (4) practical guidance, and (5) Emotional tone and empathy.

Across all models, the *accessibility of language* remains a significant challenge. Despite the advanced capabilities, the responses were found to employ complex medical jargon, posing comprehension difficulties for laypersons. This was observed highly with Bard and Bing Chat, while Google SGE and ChatGPT performed relatively better, however still inadequate. *Accuracy* varied notably among the LLMs. All models were prone to inaccuracies, Google SGE and Bing Chat were occasionally observed to share misleading medical information. Bard and ChatGPT also displayed inaccuracies, but their responses received endorsement for being comprehensive and clear.

In terms of *completeness*, LLMs showed mixed results. While Bard and ChatGPT were often recognized for their thoroughness, they sometimes missed critical details or provided excessive, overwhelming information. On the other hand, Google SGE and Bing Chat were often criticized for vague and incomplete responses, suggesting a need for a more balanced and comprehensive approach to information delivery​​. *Practical guidance* was another area of concern. LLMs also frequently failed to provide actionable and relevant advice. Google SGE's responses were noted for their vagueness, and Bing Chat's practical relevance was often questioned. Bard and ChatGPT provided some practical suggestions, but these were not consistently applicable or tailored to specific user contexts, indicating a gap in practical utility. *Emotional tone and empathy* in responses received mixed feedback. Raters appreciated attempts at empathetic communication, particularly in ChatGPT's responses, but for others the tone was either too personal or inappropriate, as seen in Bard and Bing Chat​​.

Table S1. Themes from Expert Evaluation Criteria Notes

|  | **Bard** | **Google SGE** | **Bing Chat** | **ChatGPT** |
| --- | --- | --- | --- | --- |
| **Accessibility of Language** | Many comments highlight that the language used is **too advanced** for the general public, including terms like "inherited mutations" and "lumbar puncture." There is a **need to simplify complex topics** like genetic mutations and biopsy steps using more accessible terms or even visual aids. | Assessors’ comments suggest that word choices **should be simplified** for better health literacy. One comment stated, "From a health literacy perspective, the word count is better; however, I still think the word choices could be simplified." | Some responses are described at **a grade level higher than recommended** for plain language, making it difficult for patients and families to understand. As one doctor noted, "This level of detail might be better shared in a conversation with providers." | Responses were **generally clear and well-organized**, with some being praised for hitting all major points and being easy to read. However, some responses used **advanced language and complex explanations** that would be difficult for non-medical consumers to understand without a healthcare professional's assistance. "Advanced language and explanations." "OMG. Where do I start? WAAAAY too complex" |
| **Accuracy of Information** | Assessors’ comments point out some **inaccuracies**, such as the incorrect information about urine tests detecting certain types of cancers or the inheritance of Down Syndrome. Some responses are noted to be **too focused on adult patients** when the context is pediatric cancer. There's a **need for tailored information** specific to the patient group. | **Several inaccuracies** were noted, such as "one does not inherit cancer" and "Patients who are receiving cancer medications pose no risk to children, pregnant women, or anyone else." One doctor stated, "Statement 'one does not inherit cancer' is inaccurate for some rare forms of childhood cancer." Some statements are **misleading or incorrect**, such as "The Dana-Farber Cancer Institute offers a back-to-school program" which may not apply universally. "The statement that 'most children with cancer will attend school at least some of the time...' may not be true for all." | Some responses contain **inaccuracies or irrelevant information**. For instance, "The statistics provided about cancer incidence and mortality are not related to the question asked." **Incorrect** examples, such as mentioning "prostate" in the context of pediatric oncology, were highlighted: "Inclusion of 'prostate' as an example seems inappropriate." Responses sometimes provide **misleading** information, such as, "The answer goes beyond treatment options," | Several responses contained **inaccurate or incomplete information**. "Genetic factors are poorly explained and leaves room for misinterpretation. Including prenatal exposure to diagnostic x-rays also leaves room for parents to blame themselves and has a lack of evidence." Other responses were praised for being **accurate and comprehensive**. "Comprehensive, clear, well organized, accurate." "Concise - goes through all causes. Accurate." |
| **Completeness** | There were **mixed views on the completeness** of the information provided. Several comments acknowledge that the responses are thorough and cover a wide range of relevant details: "Very well done, clearly addressing the issue that there is not one definitive answer to this question". Some comments point out missing elements essential for a complete response. For example, "Information regarding the fact that the hair will likely re-grow after treatment is missing". There seems also more than the needed information: "This offers too much detail and offers too much additional information. It would have been better if the answer focused only on signs and symptoms of anemia." | Many responses are criticized for being **too vague or incomplete**. One comment noted, "Very limited information provided, overly broad." Some responses include **extraneous information** that can confuse readers. An assessor commented, "There is added information that is unhelpful to the reader and could add confusion." | Many responses **lacked crucial details** or only **partially answered** the question. One quote highlight this: "While it gives some of the answer, it needs more specifics to provide proper information." There were sometimes **missing key details**, like staging, treatment specifics, and follow-up care. For example, "None of the choices have alluded to the need for staging," and "Does not discuss follow-up appointments and what might be done." | Many responses were praised for their **thoroughness and specificity**, providing comprehensive information. Comments included, "Thorough and provides all the information" and "Very thorough and specific. Answer the question well." Despite overall thoroughness, some responses were **missing critical information** or needed more detail. "Response does not include information about anesthesia; use of the term "modern medicine" may be offensive to some groups. "Immunotherapy is missing from Leukemia." |
| **Practical Guidance** | Practical advice was sometimes missing. One doctor advised, "probably better to make sure the reader is aware that there are multiple possible explanations," suggesting that **practical context was needed**. The responses sometimes **failed to provide practical relevance**. One comment stated, "Need to expand on some things. Just saying risk factors exist is not enough." | Responses **lack practical advice** and clear instructions. For example, "This is too vague. Talking about spleen removal for anemia without talking about why you would do that could be frightening and confuse people." Some practical advice provided is sometimes **inapplicable**. For instance, "Keep tablets whole" is not always true and can be misleading. Some responses provide practical information that may be **inappropriate** for the context. For example, "We RARELY use hormone treatment in treating children with cancer," | Some information provided was **impractical or irrelevant** to the specific context. For example, "Doesn't seem completely related to cancer. Not all answer are practical,". **Unrealistic or impractical suggestions** reduced the utility of some responses: "Doesn't seem completely related to cancer. Not all answer are practical, i.e. 'find other things to do with hands' and 'get professional help'." | **Practical suggestions** and options were appreciated. "Good lists of options, with nice to have examples", "Good answer - gives specific things that can be done. Liked the part of when to seek immediate medical care. Other answers did not discuss this" Some responses were seen as **too generic or not tailored enough** for the specific situation. "Contains some accurate information that is clearly conveyed but it is not tailored for a child receiving cancer treatment." "Accurate but generic" |
| **Emotional Tone and Empathy** | Mixed reactions to the empathetic tone in responses, with some finding it inappropriate or too personal coming from a computer. While some comments appreciate the attempt to provide emotional support, others indicate it may be **misplaced or overly personal**. "While I like the empathy this response attempted, I didn't respond well to the level of intimacy if this is coming from a bot." | Some responses **fail to provide the necessary emotional support** and reassurance that patients and their families need. For instance, assessors noted that responses were often "too medical/technical" when the questions seemed to be seeking emotional support rather than just medical facts. One comment mentioned, "Too medical/technical - the question appears to be asking for emotional support, not medical support." Furthermore, phrases like "Doctors cut out tissue" were criticized for being **too blunt**. | The use of supportive and empathetic language was occasionally seen as **unnecessary or misdirected**. For example, "I hope this helps. Let me know if you have any other questions," was seen as extraneous. The tone of some responses was critiqued **for lacking empathy or being inappropriate**. For instance, "People do not want to hear this, 'Treatment may result in remission, recurrence, or no response.'" and "The empathetic statement at the beginning of the text is unnecessary." Avoiding harsh tones was viewed as important: "Incomplete and the last section has a punitive tone." | **Empathetic tones** were appreciated for some prompts. "Good emotional support and practical things to help" Clinical tones without empathy were noted for other prompts. "The empathetic statement at the beginning of the text is unnecessary." There was a noted **lack of cultural considerations** in some responses, with one critique stating, "Information regarding different cultural considerations is missing." |
